# The stock market and NO_2_ emissions effects of COVID‐19 around the world

**DOI:** 10.1111/ecpo.12227

**Published:** 2022-08-02

**Authors:** Jens Klose, Peter Tillmann

**Affiliations:** ^1^ THM Business School Gießen Germany; ^2^ Justus‐Liebig‐University Gießen Gießen Germany

**Keywords:** COVID‐19, lockdown measures, panel VAR

## Abstract

In this paper, we study the impact of the coronavirus disease 2019 pandemic in estimated panel vector autoregression models for 92 countries. The large cross‐section of countries allows us to shed light on the heterogeneity of the responses of stock markets and nitrogen dioxide emissions as high‐frequency measures of economic activity. We quantify the effect of the number of infections and four dimensions of policy measures: (1) containment and closure, (2) movement restrictions, (3) economic support, and (4) adjustments of health systems. Our main findings show that a surprise increase in the number of infections triggers a drop in our two measures of economic activity. Propping up economic support measures, in contrast, raises stock returns and emissions and, thus, contributes to the economic recovery. We also document vast differences in the responses across subsets of countries and between the first and the second wave of infections.

## 1 INTRODUCTION

In early 2020, the coronavirus disease 2019 (COVID‐19) pandemic hit the world economy, leading to a sharp deterioration of economic activity. A part of this economic decline was due to the change in behavior of individuals in response to the virus: consumers were reluctant to travel, visit shops, and eat in restaurants. In addition, some consumers and workers were quarantined. Another source of decline was the response of governments: policy deliberately shut down large parts of public life to contain the spread of the virus. This includes restrictions on the movement and gathering of people and stay home requirements. Hence, the economic consequences of the pandemic were driven by both voluntary restraint and officially mandated lockdowns. At the same time, governments around the world also put together rescue packages to support businesses and households, and to stabilize the economy. Importantly, the timing of the spread of the virus, the policy responses, and also their economic impact differs across countries.

What are the effects of the pandemic and the subsequent containment measures on economic and financial variables? This is the question, we want to answer in this article. To do so, we quantify the impact of both the spread of the virus itself and the different facets of the policy responses for a large set of countries. As a key contribution, we estimate panel vector autoregression (VAR) models for 92 countries. Thus, we cover almost the whole world economy. The large cross‐section of countries also allows us to shed light on the heterogeneity of the responses. We classify countries according to their income level, their development status or their geographic location and estimate the models for different groups of countries. Furthermore, we take account of the fact that the responses of economic activity to governmental interventions differ across the first and the second wave of COVID‐19 infections and estimate separate wave‐specific VAR models. Importantly, our approach does not rest on the assumption that the waves are synchronized across countries.

We face three challenges when estimating the impact of the pandemic: first, we need to measure the fallout of the virus on a high frequency. Standard macroeconomic time series are available on a monthly or quarterly frequency only. Therefore, we use two variables that are available on a daily frequency for a very broad set of countries: the return on a country's stock market and the growth rate of nitrogen dioxide (NO_2_) emissions. The first reflects the response of expected future economic activity, while the latter is positively correlated with current industrial production and real gross domestic product (GDP). Over longer horizons, however, we see a negative correlation between emissions and economic growth (see, e.g., Wang & Wang, 2020) due to the transition to more sustainable economies. Yet, as we focus on the rather short period of the COVID pandemic, the negative long‐run correlation between activity and emissions should be negligible, such that the drop in production reduces emissions. In fact, we find for our sample a mean correlation of industrial production and the monthly average of our daily NO_2_ emissions of about 0.38, whereas the median correlation is even higher at about 0.49. The corresponding correlation with quarterly real GDP are 0.3 and 0.42, respectively. Second, we need to measure the variety of governmental responses. The set of indicators collected in the Oxford COVID‐19 government response tracker (Hale et al., 2021) allows us to construct four categories of the policy responses, whose impact we estimate empirically: (1) containment and closure, (2) movement restrictions, (3) economic support, and (4) adjustments of health systems. The effects of those four measures on stock prices are a priori unclear. On the one hand, tougher lockdown measures may dampen stock prices, as at least some firms can no longer apply their business model or supply chains are disrupted. On the other hand, a short lockdown can also lead to expectations of breaking a pandemic wave so that afterwards the firms can continue their existing business model. These possible two effects are also present with respect to economic support. Economic support could increase stock prices, as the government steps in to cope with at least part of the economic losses. On the other hand, also a decrease in stock prices is possible as a government intervention signals that the economic situation is actually much worse than the markets expected, which leads to additional pressure on the stock markets. Finally, also health system measures can affect stock prices either way. On the one hand, stock prices may increase as the markets belief that the introduction of new measures shortens the crisis. On the other hand, stocks may decrease as additional health measures may signal that the pandemic is even more serious than the markets believed. With respect to NO_2_ emissions, any lockdown measure in the form of closures or movement restrictions should lead to a reduction in emission, as firms cannot produce as much as they want or are restricted in delivering goods or traveling to customers. The effects of economic support or health system measures on emission is a priori undetermined.

Third, we need to identify the model,that is, we need to separate the impact of the virus itself from the consequences of the lockdown. We achieve identification through a straightforward recursive ordering of our variables. Fortunately, the nature of the pandemic lends itself to a recursive ordering: the number of infections does not contemporaneously respond to lockdown measures but needs at least 1 day. Policy, in contrast, is allowed to respond contemporaneously to the number of infections. This allows us to separate these two driving forces of economic activity.

In addition, stock returns and emissions can respond immediately to both the number of infections and changes in policy, whereas the opposite response needs at least 1 day.

Our main findings are as follows. First, economic activity is sensitive to the spread of the pandemic and the different layers of government interventions. A surprise increase in the number of infections triggers a drop in our two measures of economic activity. Both stock returns and NO_2_ emissions fall as a response to closure policies and restrictions of the movements of people. Propping up economic support measures, in contrast, raises stock returns and emissions and, thus, contributes to the economic recovery.

Second, we detect interesting cross‐sectional differences. Once we distinguish between developed and developing countries, we show that stock prices in advanced economies are more sensitive to the number of infections than in developing countries. Tightening lockdown measures reduces stock market valuations in developed countries more than in developing countries. In addition, a tighter lockdown significantly reduces emissions in developing countries. Advanced economies, in contrast, exhibit an increase in emissions following a tightening of lockdowns. We find similar differences once we split the sample along the lines of income levels or the geographical region of countries.

Third, we distinguish between the first and the second wave of infections. As a common pattern, we find that the responses of stock prices are much stronger in the first wave compared to the second. This finding pertains to the responses to the number of infections and the tightening of lockdowns. The positive impact of economic support measures found in the full sample stems from the first wave only. During the first wave, emissions fall as a response to lockdowns, but remain insensitive during the second wave. Restrictions on the movement of people during the first wave significantly reduced emissions, whereas restrictions during the second wave have no significant effect on emissions. Consistently, economic support raises emissions in the first wave, but not in the second.

Overall, our findings imply that lifting lockdowns will be expansionary, with the effect being unevenly distributed across countries. The extent of cross‐country heterogeneity should be taken into account when designing policies and making forecasts about the economic consequences of the pandemic.

The remainder of the paper is organized as follows: In Section 2, a literature review is presented. Section 3 describes in detail the dataset used. Section 4 explains our modeling framework. In Section 5, the results are presented, whereas Section 6 finally concludes.

## 2 LITERATURE REVIEW

The literature on the economic effects of the COVID pandemic is rapidly expanding. In this literature review, we discuss the papers most closely related to our research and the research gaps filled by our paper in this context. Since our paper is a global study, we focus on other studies of this kind. Therefore, we leave aside the national studies conducted with a focus on the COVID pandemic.

The first strand of literature focuses on the nexus between COVID cases or fatalities, containment measures, and economic activity. In this category, Deb et al. (2020) and Furceri et al. (2021) use a local projections framework to trace the effect of lockdowns on a range of high‐frequency indicators such as emissions, vessel trade, and extent of mobility. The intensity of lockdown is measured by the stringency index. However, the authors put the stringency index directly into the local projections. They show the effect of a change in the index itself, which is not necessarily a surprise change. Put differently, there is a large forecastable component in the stringency index, which should be taken into account. Nevertheless, both studies find that containment measures are able to dampen the spread of the virus. However, this tends to come at considerable economic costs.

Chen et al. (2020) use electricity usage and labor market indicators for the United States and Europe as proxies for economic activity and show that a spread of the pandemic and containment measures reduce economic activity, that is, lower electricity usage and raise unemployment claims. Milani (2021) uses a set of 41 countries to estimate the economic effects of COVID‐19 by employing Google Trends data with respect to the fear of unemployment. He chooses a global‐VAR framework, that is, a system of interacted single‐country VAR models. He shows that unemployment responses are very heterogeneous but tend to increase with a spread of the virus. Using data for the United States and United Kingdom, Baker, Bloom, Davis, et al. (2020) construct a COVID‐induced uncertainty index based on Baker, Bloom, and Terry (2020), and show that large parts of the contraction in economic activity can be attributed to a rise in this uncertainty.^1^ Caggiano et al. (2020) estimate the effects of a COVID‐induced uncertainty shock on the global financial cycle and industrial production in a VAR framework. They show that this shock lowers economic output and the financial cycle significantly. Feyen et al. (2021) investigate the financial sector policy response to the COVID crisis, sorting 155 jurisdictions into more or less developed economies to cope with different effects of the various groups. The authors find that politicians in richer and more populous countries have taken more stabilizing policy measures.

The most extensively investigated effect of the COVID pandemic in economics is—as a second strand of literature—the response of stock markets to the pandemic and containment measures.^2^ There are three approaches to cover stock prices on an international level in this context: first, by using some global or international stock index; second, by focusing on cross‐country comparisons; and, third, by using a panel structure to cope with the overall stock performance in a set of countries. The analysis by Dong et al. (2021) falls into the first category. The authors use the Morgan Stanley Capital International (MSCI) emerging Asia and MSCI world index in a time‐varying parameter framework, to observe changes in the estimated coefficients before and after the start of the COVID pandemic. The authors show that the response of those indices to various economic factors has changed with the beginning of the pandemic. Umar, Gubareva, et al. (2021) focus on the effects of the COVID pandemic on the MSCI environmental, social and governance leaders' indices volatility for the world, the United States, Europe, China, and emerging markets. Using a wavelet analysis, they find a high coherence between the coronavirus panic index as their measure of the spread of the virus and stock volatility. Brueckner and Vespignani (2021) focus their research on the second category of cross‐country analysis by investigating in a VAR framework the effects of the COVID pandemic on Australian and U.S. stock markets. Surprisingly they find a positive relationship between the spread of the pandemic and the stock market performance. Rehman et al. (2021) concentrate on the stock markets of the G7 countries in their wavelet analysis. They support a strong co‐movement in COVID cases and deaths with stock market returns. Conducting an event study using a sample of 63 different countries, Kapar et al. (2021) show that stock markets decline almost all over the world in response to the COVID pandemic and resulting containment measures. Davis et al. (2021) conduct a thorough analysis on the daily evolution of 35 stock markets in the wake of the COVID pandemic. They show that stock prices first dropped and recovered later. However, there are three exceptions from this overall pattern: China, South Korea and Taiwan.

2.1

A panel analysis with respect to stock markets is conducted by Alexakis et al. (2021). They investigate the effects of COVID cases and containment measures, as well as country spillovers on 45 stock markets, and find evidence of negative spillover effects from containment measures. Chatjuthamard et al. (2021) employ a panel with 43 different stock indices and verify that the growth rate of COVID cases significantly reduces the stock market performance. Heyden and Heyden (2020) use a panel of U.S. and European stocks to conduct an event analysis on the effects of the arrival of COVID in a country and the first policy responses on those stocks. They find that the announcement of the first COVID cases as well as fiscal policy measures lead to a drop in stock prices, whereas the reverse is true for the first monetary policy annoucement. Klose and Tillmann (2021) also use an event study for 29 European stock market indices and evaluate the effects of COVID cases, as well as monetary and national or European fiscal support measures taken. They show that the alternative policy measures taken have very different effects on stock prices. Shafiullah et al. (2021) turn this analysis upside down by investigating whether the drop in stock markets can predict the size of the economic stimulus packages in times of the COVID pandemic. They are able to show that a larger decline in stock prices is associated with larger stimulus packages in richer countries, and that monetary policy tends to be more responsive than fiscal policy. Conceptually closest to our analysis is Zhou and Kumamoto (2020), who use a panel of 15 countries to investigate the effects of the COVID pandemic and containment measures using a panel VAR. They find that stricter containment measures lead to a decline in stock prices. However, the approach in this paper focuses on a larger set of countries, for a longer sample period and a more detailed breakdown of containment measures.

To evaluate the economic effects of the COVID pandemic and of containment measures, we need high frequency data. One of these variables is the emission of greenhouse gases, as those (at least in the short term) should be higher if companies are producing and people are traveling to work. Therefore, we use emission as an indicator of economic activity. The effects of COVID pandemic on emissions have been investigated extensively in environmental science. Most of these studies concentrate on CO_2_‐emissions and find for various countries or cities that emissions decline during the COVID pandemic (Adhikari et al., 2021; Hoang et al., 2021; Kumar et al., 2022; Ray et al., 2021; Schulte‐Fischedick et al., 2021). Those find that the COVID pandemic has reduced CO_2_ emissions. Some articles focus on a broader set of emission gases. Gettelman et al. (2021) use a forecasting model on the period of the COVID pandemic to investigate the effects of a broad set of greenhouse gases on the climate. Yang et al. (2021) perform a meta study for various countries or regions with respect to a variety of emission gases before and after the start of the COVID pandemic. They find that emissions of CO_2_ and NO_2_ are reduced due to the COVID crisis. Zhang et al. (2021) use satellite data for NO_2_ emissions and match this with lockdown measures in 187 countries or jurisdictions. They find that stricter lockdown measures are associated with less emissions. Even though not a cross‐country study, Asna‐ashary et al. (2020) estimate in a panel VAR for Iranian regions the effects of the COVID pandemic on air pollution measured by particulate matter 2.5. They find supportive evidence for a negative response of pollution to the spread of the virus.

To the best of our knowledge, there is currently only the paper by Mzoughi et al. (2020) dealing with the consequences of the COVID pandemic on stock markets (here measured as stock market volatility) and emissions (measured as CO_2_ emissions) simultaneously. They use global data in a VAR framework and find that CO_2_ emissions fall and stock market volatility increases once the COVID infections rise, although their results appear to be hardly significant.

This paper contributes to the literature in several ways: first, we employ more detailed data on containment measures by building four different groups (closures, movement restrictions, economic support, and health systems), thus being able to identify differences between policies. Second, we rely on a larger set of 92 countries all over the world and thus way more than most of the studies mentioned above. Therefore, we have the opportunity to not only investigate the overall (global) effects, but also subdivide our sample geographically and with respect to the development status. Third, we rely on an extended sample period compared with the other studies. We have the advantage of being able to identify different waves of the pandemic in the countries and thus possible differences among the response to the COVID cases and containment policies in the various waves. Finally, we focus on financial and real activity variables separately to investigate whether there are differences in their reactions to the COVID pandemic and containment measures.

## 3 THE DATASET

In this section, we explain the construction of the variables for our panel VAR analysis. As we are interested in the financial and real impact of the COVID pandemic and the underlying policy responses, we use a daily frequency as COVID cases or deaths are typically reported on a day‐to‐day basis. We approximate the financial response by the evolution of the leading stock indices of the sample countries. The real effects are approximated by the emission of NO_2_ as it is a byproduct of the combustion of fossil fuels, that is, resulting from energy production and mobility.^3^ Thus, we expect a positive correlation between NO_2_ emissions and economic activity as, for example, more energy is needed for production purposes and more people travel to work. The variable covering the severity of the pandemic is the number the reported COVID cases. We use those instead of the alternative of reported COVID deaths as the cases are typically seen as leading indicator, that is, the more people are currently infected, the higher the number of deaths in about 1–2 weeks.

### 3.1 Policy response variables

Our main contribution is an analysis of the effects of policy changes due to the COVID pandemic on financial and real variables in a large panel of countries. To capture these policy changes, we rely on the data of the University of Oxford COVID‐19 government response tracker (Hale et al., 2021). We use all ordinally measured variables as those are directly comparable across countries. Hale et al. (2021) cluster those variables into three groups: (1) containment and closure, (2) economic response, and (3) health systems. We follow this categorization but divide the containment and closure measures into two subcategories representing closures on the one hand and movement restrictions on the other hand. Hence, we work with four categories of policy responses: (1) containment and closure, (2) movement restrictions, (3) economic support, and (4) health systems.

The closure category contains four different policy measures. The same is true for the movement category. The economic response category comprises two policy measures and the health system category summarizes six policy measures. A detailed description of the different measures based on Hale et al. (2021) and the ordinal steps is presented in Table 1. All indicators are ordinally scaled, where a value of 0 means that the measure is not implemented at all and the highest number reflects the strictest implementation of a certain measure. The highest realization of each indicator may differ from measure to measure. Moreover, an additional 0/1 variable is introduced for certain measures signaling whether the measure was targeted either geographically, with respect to a specific sector or costs. So, for example, if the measure was geographically targeted to a certain region in a country on a certain day only, the value of the index would be 0. If it was a general measure applied throughout the whole country, it would be 1 instead.

**Table 1:** Government response indicator

Indicator	Description	Ordinal Steps	General or Targeted Measure
Closure measures			
School closing	Closing of schools and universities	0 = No measure	Geographical
		1 = Recommend closing, or all schools open with alterations	0 = Targeted
		2 = Require closing some levels	1 = General
		3 = Require closing all levels	
Workplace closing	Closings of workplaces	0 = No measure	Geographical
		1 = Recommend closing, or work from home	0 = Targeted
		2 = Require closing some sectors	1 = General
		3 = Require closing all but essential sectors	
Cancel public events	Canceling public events	0 = No measure	Geographical
		1 = Recommend canceling	0 = Targeted
		2 = Require canceling	1 = General
Restrictions on gatherings	Cutoff size for bans on gatherings	0 = No restrictions	Geographical
		1 = Restrictions > 1000 people	0 = Targeted
		2 = Restrictions 101–1000 people	1 = General
		3 = Restrictions 11–100 people	
		4 = Restrictions < 10 people	
Movement measures			
Close public transport	Closing of public transport	0 = No measure	Geographical
		1 = Recommend closing or reduced volume, route, availability	0 = Targeted
		2 = Require closing	1 = General
Stay at home requirements	Orders to “shelter in place” and otherwise confine at house	0 = No measure	Geographical
		1 = Recommend not leaving home	0 = Targeted
		2 = Require not leaving house with exceptions	1 = General
		3 = Require not leaving house with minimal exceptions	
Restrictions on internal movement	Restrictions on internal movement	0 = No measure	Geographical
		1 = Recommend not to travel between regions and cities	0 = Targeted
		2 = Internal movement restrictions in place	1 = General
International travel controls	Restrictions on international travel	0 = No measure	
		1 = Screening	
		2 = Quarantine arrivals from high‐risk regions	
		3 = Ban of arrivals from some regions	
		4 = Ban on all regions or total border closure	
Economic response measures			
Income support	Government covering salaries or	0 = No income support	Sectoral
	providing direct cash payments,	1 = Less than 50% replacement	0 = Only formal sector
	universal basic income	2 = More than 50% replacement	1 = Also informal sector
Debt/contract relief	Government freezing financial obligations	0 = No relief	
		1 = Narrow relief	
		2 = Broad relief	
Health systems measures			
Public information campaigns	Presence of public information campaigns	0 = No campaign	Geographical
		1 = Public officials urging caution about COVID‐19	0 = Targeted
		2 = Coordinated public information campaign	1 = General
Testing policy	Testing strategies	0 = No testing policy	
		1 = Only to those who have symptoms and meet specific criteria	
		2 = Anyone with symptoms	
		3 = Testing for everyone	
Contact tracing	Use of measure to trace contacts	0 = No contact tracing	
		1 = Limited contact tracing (not for all cases)	
		2 = Comprehensive contact tracing (for all cases)	
Facial coverings	Policies of facial coverings outside home	0 = No policy	Geographical
		1 = Recommended	0 = Targeted
		2 = Required in some situations	1 = General
		3 = Required all public places with other people present or all situations when social distancing is impossible 4 = equired outside home	
Vaccination policy	Policies for vaccine delivery to different groups	0 = No availability	Costs
		1 = Available to one of the following groups: Key workers, vulnerable groups, elderly groups	0 = individual cost
		2 = Available to two of the following groups: Key workers, vulnerable groups, elderly groups 3 = Available to all of the following groups: Key workers, vulnerable groups, elderly groups 4 = Available to the three groups above plus partial additional availability 5 = Universal availability	1 = no or minimal individual costs
Protection of elderly people	Policies to protect elderly people	0 = No measure 1 = Recommended isolation, hygiene and visitor restrictions in LTCF or elderly people to stay at home 2 = Narrow restrictions for isolation, hygiene and visitor restrictions in LTCF or elderly people to stay at home 3 = Extensive restrictions for isolation, hygiene and visitor restrictions in LTCF or elderly people to stay at home	

*Note*: Indications and descriptions based on Hale et al. (2021).

Abbreviation: LTCF, Long Term Care Facilities.

To guarantee that each measure has the same importance in our four groups, we calculate variables ranging from 0 (the measure is not implemented at all) to 100 (the measure is implemented in its strictest way) in line with recommendations of Hale et al. (2021). This means that for the measures with ordinal steps only but without a general or targeted indication, the variables are calculated as

(1) $$x_{it}=100\times \frac{m_{it}}{M}.$$

In Equation (1), *x*_*it*_ is the variable ranging between 0 and 100 of country *i* at day *t*, whereas *m*_*it*_ is the realization of the measure as presented in Table 1 for the very same country on the same day, and *M* stands for the maximum realization of the measure, which is constant over countries and time. If the measure is flagged as a general or targeted 0/1‐indicator, the variables are computed according to

(2) $$x_{it}=100\times \frac{m_{it}-0.5(1-g_{it})}{M}.$$

In this equation, *g*_*it*_ is the general or targeted indicator of a certain country on a certain day, which could be 0 or 1. This adjustment guarantees that in case of a targeted action, there is a discount of 0.5 to the ordinal measure. By construction, in case of no actions taken (thus, *m*_*it*_ being zero), *g*_*it*_ = 1, to guarantee that *x*_*it*_ cannot fall into negative territory.

Finally, the different variables calculated with the formulas above are merged into the four different groups by using the arithmetic mean in line with the calculations of, for example, the stringency index in Hale et al. (2021), which does, however, differ in its composition from our groups.

The series on COVID cases, stock prices and NO_2_ emissions are rather volatile. Therefore, we calculate 5‐day moving averages of percentage growth rates to smooth them. To correspond with those 5‐day moving averages, we apply the same procedure to the changes in the four policy intervention categories.

As we focus on the period of the COVID pandemic, our sample period starts on January 31, 2020. The speed by which the COVID pandemic spread differed across continents and countries. Although some countries already struggled to contain the pandemic, others were still untouched. As a consequence, our panel is unbalanced. For every country, the sample begins with the first occurrence of the virus. The end of the sample is uniformly the 16th April 2021.^4^ Within this period, most countries faced several waves of the COVID‐pandemic. In total, our panel covers 92 countries, see Table 2. For those countries, we were able find stock prices or data on NO_2_ emissions or both. The number of COVID cases, as well as the policy measures are available for all 92 countries. Summary statistics and panel unit root tests using the tests of Levin et al. (2002) and Im et al. (2003) are presented in Table 3. For all our variables, we can reject the hypothesis of a unit root.

**Table 2:** Country sample and data availability

Country	Stock prices	NO_2_ emission	Policy measures	Country	Stock prices	NO_2_ emission	Policy measures
Argentina	X	X	X	Luxembourg	X	X	X
Australia	X	X	X	Malaysia	X		X
Austria	X	X	X	Malta	X		X
Bangladesh	X		X	Mauritius	X		X
Belgium	X	X	X	Mexico	X	X	X
Bolivia		X	X	Mongolia		X	X
Bosnia and Herzegovina	X	X	X	Morocco	X		X
Brazil	X	X	X	Netherlands	X	X	X
Bulgaria	X	X	X	New Zealand	X	X	X
Cambodia	X		X	Nigeria	X		X
Canada	X	X	X	Norway	X	X	X
Chile	X	X	X	Oman	X		X
China	X	X	X	Pakistan	X		X
Colombia	X	X	X	Panama	X		X
Croatia	X	X	X	Peru	X	X	X
Cyprus	X	X	X	Philippines	X	X	X
Czechia	X	X	X	Poland	X	X	X
Denmark	X	X	X	Portugal	X	X	X
Ecuador	X		X	Qatar	X		X
Egypt	X		X	Romania	X	X	X
Estonia	X	X	X	Russia	X	X	X
Finland	X	X	X	Saudi Arabia	X	X	X
France	X	X	X	Serbia	X	X	X
Georgia	X	X	X	Singapore	X		X
Germany	X	X	X	Slovakia	X	X	X
Ghana	X		X	Slovenia	X		X
Greece	X	X	X	South Africa	X	X	X
Hong Kong	X	X	X	South Korea	X	X	X
Hungary	X	X	X	Spain	X	X	X
Iceland	X	X	X	Sri Lanka	X		X
India	X	X	X	Sweden	X	X	X
Indonesia	X		X	Switzerland	X	X	X
Iran		X	X	Taiwan	X	X	X
Ireland	X	X	X	Tanzania	X		X
Israel	X	X	X	Thailand	X	X	X
Italy	X	X	X	Tunisia	X		X
Jamaica	X		X	Turkey	X	X	X
Japan	X	X	X	Uganda	X		X
Jordan		X	X	Ukraine	X		X
Kazakhstan		X	X	United Arab Emirates	X	X	X
Kenya	X		X	United Kingdom	X	X	X
Kosovo		X	X	United States of America	X	X	X
Kuwait	X	X	X	Venezuela	X		X
Latvia	X		X	Vietnam	X	X	X
Lebanon	X		X	Zambia	X		X
Lithuania	X		X	Zimbabwe	X		X

*Note*: X marks availability of at least some variables for the country.

**Table 3:** Descriptive statistics and panel unit tests

	Stock	NO_2_	COVID cases	Closure	Movement	Econ. support	Health
Descriptive statistics							
Mean	0.054	0.632	3.765	0.204	0.134	0.152	0.213
Minimum	−9.247	−93.369	−18.449	−15.000	−13.750	−20.000	−5.889
Maximum	13.251	960.000	107.721	20.000	20.000	20.000	12.222
SD	0.896	14.349	8.103	2.107	1.785	1.705	0.920
Panel unit root test							
Levin et al. (2002)	−39.067 (.000)	−95.231 (.000)	−68.655 (.000)	−49.259 (.000)	−54.884 (.000)	−43.311 (.000)	−62.839 (.000)
Im et al. (2003)	−41.062 (.000)	−88.295 (.000)	−66.900 (.000)	−46.332 (.000)	−48.347 (.000)	−40.025 (.000)	−53.517 (.000)

*Note*: Panel unit root test testing for a common unit root as introduced by Levin et al. (2002) or for an individual unit root as introduced by Im et al. (2003). For the unit root tests, we show the corresponding *p* in parenthesis.

Although we estimate a panel VAR with all 92 countries jointly, we also differentiate the sample to shed light on the differences across country groups. We implement this differentiation in three dimensions. The categorization of countries to different groups is shown in Tables 4, 5, and 6. First, the countries are grouped according to their development status. The classification used in this context follows United Nations (2020). We group the countries into developed, transition and developing countries. Second, the countries are grouped with respect to their income level following the definition of the World Bank.^5^ According to this classification, the countries are sorted into one of the following four categories: high‐income, upper‐middle‐income, lower‐middle‐income, or low‐income countries. The third differentiation is geographical. Here the countries are associated with their continent, that is, Africa, Asia, Europe, North and Middle America, South America, or Australia.

**Table 4:** Classification developed versus developing countries

Country	Developed	Transition	Developing	Country	Developed	Transition	Developing
Argentina			X	Luxembourg	X		
Australia	X			Malaysia			X
Austria	X			Malta	X		
Bangladesh			X	Mauritius			X
Belgium	X			Mexico			X
Bolivia			X	Mongolia			X
Bosnia and Herzegovina		X		Morocco			X
Brazil			X	Netherlands	X		
Bulgaria	X			New Zealand	X		
Cambodia			X	Nigeria			X
Canada	X			Norway	X		
Chile			X	Oman			X
China			X	Pakistan			X
Colombia			X	Panama			X
Croatia	X			Peru			X
Cyprus	X			Philippines			X
Czechia	X			Poland	X		
Denmark	X			Portugal	X		
Ecuador			X	Qatar			X
Egypt			X	Romania	X		
Estonia	X			Russia		X	
Finland	X			Saudi Arabia			X
France	X			Serbia		X	
Georgia		X		Singapore			X
Germany	X			Slovakia	X		
Ghana			X	Slovenia	X		
Greece	X			South Africa			X
Hong Kong			X	South Korea			X
Hungary	X			Spain	X		
Iceland	X			Sri Lanka			X
India			X	Sweden	X		
Indonesia			X	Switzerland	X		
Iran			X	Taiwan			X
Ireland	X			Tanzania			X
Israel			X	Thailand			X
Italy	X			Tunisia			X
Jamaica			X	Turkey			X
Japan	X			Uganda			X
Jordan			X	Ukraine		X	
Kazakhstan		X		United Arab Emirates			X
Kenya			X	United Kingdom	X		
Kosovo		X		United States of America	X		
Kuwait			X	Venezuela			X
Latvia	X			Vietnam			X
Lebanon			X	Zambia			X
Lithuania	X			Zimbabwe			X

*Note*: X marks the classification of a country into a category. Classification according to United Nations (2020).

**Table 5:** Classification via income levels

Country	High	Upper‐middle	Lower‐middle	Low	Country	High	Upper‐middle	Lower‐middle	Low
Argentina		X			Luxembourg	X			
Australia	X				Malaysia		X		
Austria	X				Malta	X			
Bangladesh			X		Mauritius		X		
Belgium	X				Mexico		X		
Bolivia			X		Mongolia			X	
Bosnia and Herzegovina		X			Morocco			X	
Brazil		X			Netherlands	X			
Bulgaria		X			New Zealand	X			
Cambodia			X		Nigeria			X	
Canada	X				Norway	X			
Chile	X				Oman	X			
China		X			Pakistan			X	
Colombia		X			Panama	X			
Croatia	X				Peru		X		
Cyprus	X				Philippines			X	
Czechia	X				Poland	X			
Denmark	X				Portugal	X			
Ecuador		X			Qatar	X			
Egypt			X		Romania		X		
Estonia	X				Russia		X		
Finland	X				Saudi Arabia	X			
France	X				Serbia		X		
Georgia		X			Singapore	X			
Germany	X				Slovakia	X			
Ghana			X		Slovenia	X			
Greece	X				South Africa		X		
Hong Kong	X				South Korea	X			
Hungary	X				Spain	X			
Iceland	X				Sri Lanka		X		
India			X		Sweden	X			
Indonesia			X		Switzerland	X			
Iran		X			Taiwan	X			
Ireland	X				Tanzania				X
Israel	X				Thailand		X		
Italy	X				Tunisia			X	
Jamaica		X			Turkey		X		
Japan	X				Uganda				X
Jordan		X			Ukraine			X	
Kazakhstan		X			United Arab Emirates	X			
Kenya			X		United Kingdom	X			
Kosovo		X			United States of America	X			
Kuwait	X				Venezuela		X		
Latvia	X				Vietnam			X	
Lebanon		X			Zambia			X	
Lithuania	X				Zimbabwe			X	

*Notes*: X marks the classification of a country into a category. Classification according to World Bank.

**Table 6:** Geographical classification

Country	Africa	Asia	Europe	N.‐M. America	S. America	Australia	Country	Africa	Asia	Europe	N.‐M. America	S. America	Australia
Argentina					X		Luxembourg			X			
Australia						X	Malaysia		X				
Austria			X				Malta			X			
Bangladesh		X					Mauritius	X					
Belgium			X				Mexico				X		
Bolivia					X		Mongolia		X				
Bosnia and Herzegovina			X				Morocco	X					
Brazil					X		Netherlands			X			
Bulgaria			X				New Zealand						X
Cambodia		X					Nigeria	X					
Canada				X			Norway			X			
Chile					X		Oman		X				
China		X					Pakistan		X				
Colombia					X		Panama				X		
Croatia			X				Peru					X	
Cyprus			X				Philippines		X				
Czechia			X				Poland			X			
Denmark			X				Portugal			X			
Ecuador					X		Qatar		X				
Egypt	X						Romania			X			
Estonia			X				Russia			X			
Finland			X				Saudi Arabia		X				
France			X				Serbia			X			
Georgia		X					Singapore		X				
Germany			X				Slovakia			X			
Ghana	X						Slovenia			X			
Greece			X				South Africa	X					
Hong Kong		X					South Korea		X				
Hungary			X				Spain			X			
Iceland			X				Sri Lanka		X				
India		X					Sweden			X			
Indonesia		X					Switzerland			X			
Iran		X					Taiwan		X				
Ireland			X				Tanzania	X					
Israel		X					Thailand		X				
Italy			X				Tunisia	X					
Jamaica				X			Turkey			X			
Japan		X					Uganda	X					
Jordan		X					Ukraine			X			
Kazakhstan		X					United Arab Emirates		X				
Kenya	X						United Kingdom			X			
Kosovo			X				United States of America				X		
Kuwait		X					Venezuela					X	
Latvia			X				Vietnam		X				
Lebanon		X					Zambia	X					
Lithuania			X				Zimbabwe	X					

*Note*: X marks the classification of a country into a category.

Abbreviations: N.‐M. America, North and Middle America; S. America, South America.

### 3.2 Pandemic waves

In most countries, the pandemic spread in waves. We evaluate whether the responses to stock prices and NO_2_ emissions differ across the successive waves of COVID infections.^6^ Importantly, these waves were not synchronized across countries such that we cannot implement a simple sample split.

To identify the waves, we slightly modify a classification algorithm introduced by the British Office for National Statistics (2021).^7^ We rely on three variables to measure waves. First, the daily growth rate of new infections. To account for the weekly cyclicality in some countries, we use the smoothed series of new cases series provided by *Our World in Data* to calculate the daily growth rates. Second, the reproduction rate (*R*) measures how many people an infected person infects on average. Third, the positivity rate measuring the percentage of positive COVID‐19 tests.

The daily growth rate of COVID infections and the *R* rate determine the start of a wave. A wave begins if two criteria are met simultaneously: first, the daily growth rate is positive for ten weekdays in a row (i.e., for day *t* and the nine preceding weekdays).^8^ Second, the *R* rate needs to exceed unity in order for a wave to start meaning that one infected person infects more than one other person and, thus, the pandemic spreads. The end of a wave is determined by the positivity rate. If the positivity rate falls to the lowest quantile over all observations of each country, a wave is supposed to have ended. With this approach, we are well in line with the definition of the British Office for National Statistics (2021), who defines a wave to end if the positivity rate falls below 0.1% in England. We come up with an almost similar threshold for the United Kingdom at a slightly different time period. However, our quantile approach has the advantage of identifying wave ends for other countries, that is, even those that face constantly higher positivity rates than the United Kingdom.

Using this approach, we are able to identify the waves for each country as shown in Table 7. Due to missing data of at least 1 of our indicator variables, we had to delete 16 countries from the analysis. Moreover, for three countries, no waves could be detected. For the large majority of the remaining countries, we detect two COVID waves. This holds for 45 out of the remaining 73 countries. For 27 countries, we detect only 1 wave and only 1 country faced 3 waves (the United States).^9^ Note that the last wave often ends with the end of our sample period. Thus it has to be assumed that the wave continues beyond the end of our sample period.

**Table 7:** COVID‐wave classification

Country	First wave	Second wave	Comments	Country	First wave	Second wave	Comments
Argentina	05/14/20–02/16/21	04/05/21–04/16/21		Luxembourg	03/10/20–05/25/20	10/07/20–04/16/21	
Australia	03/13/20–05/11/20	06/22/20–09/25/20		Malaysia	12/30/20–04/16/21		
Austria	03/03/20–05/27/20	08/10/20–04/16/21		Malta	10/02/20–04/16/21		
Bangladesh	04/03/20–02/04/21	03/03/21–04/16/21		Mauritius			Missing data
Belgium	03/13/20–05/01/20	10/01/20–04/16/21		Mexico	04/06/20–03/26/21		
Bolivia	05/04/20–11/06/20	12/08/20–04/09/21		Mongolia	11/09/20–12/01/20	01/27/21–04/16/21	
Bosnia and Herzegovina	06/19/20–01/07/21	03/10/21–04/16/21		Morocco	03/13/20–05/22/20	10/06/20–04/16/21	
Brazil			Missing data	Netherlands			Missing data
Bulgaria	10/02/20–04/16/21			New Zealand	03/16/20–05/01/20		
Cambodia			Missing data	Nigeria	12/03/20–03/12/21		
Canada	03/11/20–06/29/20	09/16/20–04/16/21		Norway	03/03/20–06/10/20	10/19/20–04/16/21	
Chile	03/20/20–11/06/20	01/07/21–04/16/21		Oman			Missing data
China			Missing data	Pakistan	03/09/20–08/27/20	11/02/20–04/16/21	
Colombia	03/13/20–02/12/21	03/10/21–04/16/21		Panama	11/19/20–03/09/21		
Croatia	03/13/20–05/01/20	08/10/20–04/16/21		Peru	07/13/20–10/22/20		
Cyprus	10/07/20–04/16/21			Philippines	02/22/21–04/16/21		
Czechia	03/13/20–03/24/21			Poland	03/10/20–05/18/20	07/21/20–04/16/21	
Denmark	03/23/20–06/11/20	09/02/20–03/23/21		Portugal	03/19/20–05/04/20	08/26/20–03/17/21	
Ecuador			No wave detected	Qatar	04/15/20–10/13/20	01/15/21–04/16/21	
Egypt			Missing data	Romania	03/05/20–05/11/20	07/03/20–04/16/21	
Estonia	09/16/20–04/16/21			Russia	03/12/20–08/07/20	09/28/20–04/16/21	
Finland	09/30/20–04/16/21			Saudi Arabia	04/09/20–11/20/20	02/01/21–04/16/21	
France	03/13/20–05/28/20	08/03/20–04/16/21		Serbia	03/27/20–08/31/20	10/05/20–04/16/21	
Georgia			Missing data	Singapore	03/13/20–10/05/20		
Germany			Missing data	Slovakia	09/23/20–04/16/21		
Ghana			No wave detected	Slovenia	08/11/20–04/16/21		
Greece	06/30/20–07/28/20	10/12/20–04/16/21		South Africa	05/07/20–04/16/21		
Hong Kong			Missing data	South Korea	02/20/20–04/17/20	08/07/20–04/16/21	
Hungary	03/13/20–06/05/20	08/26/20–04/16/21		Spain	02/26/20–04/29/20	07/09/20–04/16/21	
Iceland	03/05/20–05/04/20			Sri Lanka	11/24/20–04/16/21		
India	03/12/20–12/30/20	02/25/21–04/16/21		Sweden	03/20/20–08/27/20	09/18/20–04/16/21	
Indonesia	03/13/20–04/29/20	08/25/20–03/26/21		Switzerland	08/10/20–04/16/21		
Iran	05/05/20–06/25/20	10/14/20–04/16/21		Taiwan			No wave detected
Ireland	04/02/20–06/11/20	09/24/20–04/16/21		Tanzania			Missing data
Israel	05/29/20–04/05/21			Thailand	03/10/20–05/14/20	04/05/21–04/16/21	
Italy	02/24/20–06/09/20	09/18/20–04/16/21		Tunisia			Missing data
Jamaica	03/01/21–04/16/21			Turkey	03/17/20–08/21/20	11/06/20–04/16/21	
Japan	03/20/20–05/11/20	11/05/20–04/16/21		Uganda	08/07/20–04/16/21		
Jordan	09/07/20–01/15/21	02/22/21–04/16/21		Ukraine	03/19/20–05/13/20	07/23/20–04/16/21	
Kazakhstan	04/10/20–04/24/20	10/26/20–04/16/21		United Arab Emirates	03/27/20–04/16/21		
Kenya	03/08/21–04/16/21			United Kingdom	02/24/20–07/02/20	07/23/20–04/08/21	
Kosovo			Missing data	United States of America	03/02/20–05/29/20	06/22/20–09/10/20	Third wave detected 10/13/20–03/10/21
Kuwait	04/16/20–12/02/20	01/27/21–04/16/21		Venezuela			Missing data
Latvia	03/13/20–05/29/20	10/08/20–04/16/21		Vietnam			Missing data
Lebanon			missing data	Zambia	12/17/20–04/16/21		
Lithuania	03/13/20–05/12/20	09/16/20–04/16/21		Zimbabwe	12/29/20–03/05/21		

*Note*: Missing data signals that at least one of the three variables needed for the computation of the waves is not available or there are too few observations to compute meaningful results.

In the empirical analysis below, we estimate the panel VAR model separately for the first and the second COVID waves.

## 4 MODEL

We estimate a panel VAR model to capture the dynamics of the variables. This class of models is particularly attractive for our purpose because it allows us to estimate the dynamic effect of lockdown shocks for a large set of countries. Our model is given by

(3) $${\text{Ay}}_{\mathrm{it}}=d_{i}+F_{1}y_{\mathrm{it}-1}+\text{…}+F_{s}y_{\mathrm{it}-s}+\varepsilon_{\mathrm{it}},$$

with *s* lags for country *i* = 1,…,*N* and time *t* = *s* + 1,….,*T*, where the *n* × 1 vector **y**_*it*_ contains the endogenous variables. The *n* × 1 vector **d**_**i**_ collects the country fixed‐effects and the *n* × *n* matrices **A** and **F**_**1**_,…,**F**_**s**_ contain the VAR coefficients.

The structural shocks are in *ε*_*it*_ with *ε*_*it*_ ∼ *N* (0,**ΣΣ** ^0^). The 3 × 1 vector of endogenous variables is

(4) $$y^{\mathrm{it}}=[{\mathrm{cases}}_{it}\;{\mathrm{indi}}_{it}^{j}\;y_{it}].$$

The number of new COVID‐19 infections is *cases*_*it*_, whereas the category *j* of the policy response indicator is denoted by *indi* ^*j*^_*it*_ ranging from 1 to 4. The third endogenous variable in the VAR, *y*_*it*_, is either the daily stock return or the level of NO_2_ emissions in country *i*. To keep the VAR model as compact as possible, we use either stock returns or emissions as our third variable. We impose the restriction that the autoregressive coefficients are identical across countries. Below, we shed light on the strictness of this assumption by distinguishing between groups of countries. The estimated VAR includes two lags of the endogenous variables.

Since we are interested in the causal effect of lockdown shocks on the other endogenous variables, we need to impose identifying restrictions onto the VAR model. We chose a recursive identification scheme, which amounts to imposing an order on the contemporaneous interaction among the variables. We assume that **A** is lowertriangular. Premultiplying the VAR model with **A** ^−1^ recovers the reduced‐form model

(5) $$y_{it}=c_{i}+B_{1}y_{it-1}+\text{…}+B_{s}y_{it-s}+A^{-1}\sum \varepsilon_{it},$$

with *ε*_*it*_ ∼ *N* (0,**I**_*k*_), where **c**_*i*_ = **A** ^−**1**^ **d**_*i*_ and **B**_*j*_ = **A** ^−**1**^ **F**_*j*_. **Σ** is an *n* × *n* matrix with SDs on the main diagonal.

Fortunately, the nature of the variables lends itself to a straightforward ordering: We assume that the number of infections responds with a lag of at least 1 day to a tightening or easing of lockdowns as reflected in the change of the policy response indicator. Policymakers can, in contrast, respond contemporaneously to a change in the number of infections. Hence, the COVID cases are ordered first and the policy response subcomponent is ordered second. The third variable can respond contemporaneously to either the policy response index or the number of COVID cases, whereas these two variables need at least 1 day to respond to changes in the third variable. We believe this recursive scheme to be an innocuous constraint.

## 5 RESULTS

We present the estimates in terms of impulse response functions. In each figure, we show the response of the third variable, the variables whose responses we are mostly interested in, to a shock in the number of COVID cases or the subcomponent *j* of the policy response index. A shock is an unexpected change in either of these variables, for example, a surprise tightening of lockdowns or an unexpected increase in the number of COVID cases. Each figure also shows the 95% confidence band around the estimated impulse responses.

### 5.1 Worldwide results

**Figure 1:**
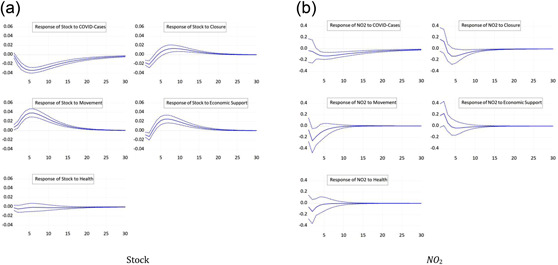
Reaction full sample. Impulse responses of stock prices (left) and nitrogen dioxide (NO_2_) emissions (right) to a one‐standard‐deviation shock in coronavirus disease (COVID) cases and the four indicators of government responses. The dashed lines indicate the 95% confidence interval. [Color figure can be viewed at wileyonlinelibrary.com]

Figure 1 (left) depicts the response of stock prices to the five shocks we consider, an increase in the number of cases or an increase in one of the four subcomponents of the policy response index. Stock prices fall significantly after a shock to the number of COVID cases. This response, such as most other responses, is highly statistically significant. The peak response occurs 5 days after the shock. The finding is in line with those of Alexakis et al. (2021), Chatjuthamard et al. (2021), Davis et al. (2021), Heyden and Heyden (2020), Kapar et al. (2021), Mzoughi et al. (2020), or Rehman et al. (2021). A tightening of lockdowns also reduces the valuation of the stock market. Stock returns fall by 0.02% points after an increase in the closure component of the index by 1 SD, but recover after about 5 days. This result is comparable to the ones of Alexakis et al. (2021), Kapar et al. (2021), or Zhou and Kumamoto (2020). If authorities extend economic support to the economy as reflected by an unexpected increase in the economic support subcomponent, stock prices strongly recover after about 3 days, while they initially fall, possibly due to negative news given with the support measure that the crisis is more severe. These responses are all consistent with our economic intuition and in line with literature (Heyden & Heyden, 2020; or Klose & Tillmann, 2021). The response of stock prices to a tightening in the restrictions of movements, in contrast, is puzzling. Stock prices appreciate after such a tightening, which could be explained based on the notion that a restriction of movements is considered an effective containment of the spread of the virus, which raises expected future economic conditions. The stock markets seem insensitive to changes in the healthdimension of the policy response index. This finding prevails in all other impulse response functions.

Figure 1 (right) reports the responses of NO_2_ emissions. A surprise increase in the number of infections reduces the level of emissions. With this finding we are in line with Adhikari et al. (2021), Asna‐ashary et al. (2020), Gettelman et al., (2021), Hoang et al. (2021), Kumar et al. (2022), Mzoughi et al. (2020), Ray et al. (2021), Schulte‐Fischedick et al. (2021), or Yang et al. (2021). Likewise, a stricter closure policy or a tightening of restrictions to the movement of people lead to a significant fall in emissions in line with Zhang et al. (2021). Since emissions closely reflect economic activity such as industrial production and transportation, these results show the large economic cost of lockdowns. More generous economic support, in contrast, tends to increase emissions. Hence, economic support is effective in containing the economic costs of the pandemic.

### 5.2 Developed versus developing countries

**Figure 2:**
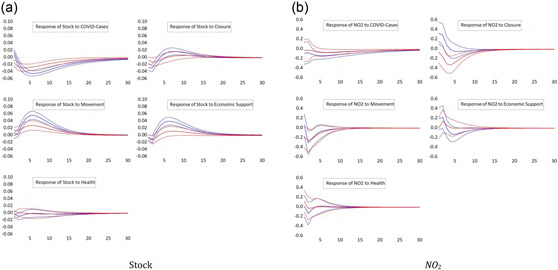
Reaction for differing development status. Impulse responses of stock prices (left) and nitrogen dioxide (NO_2_) emissions (right) to a one‐standard‐deviation shock in coronavirus disease (COVID) cases and the four indicators of government responses. The dashed lines indicate the 95% confidence interval. The blue lines are the responses for developed countries, whereas the red lines give the responses of developing countries. The classification of countries follows United Nations (2020). [Color figure can be viewed at wileyonlinelibrary.com]

We now differentiate between countries on the basis of their level of development. We estimate the panel VAR model separately for developed countries, as well as developing countries, as explained in the previous section.^10^ Figure 2 (left) shows the estimated impulse responses of stock prices. In advanced economies, stock prices are more sensitive to the number of COVID cases compared with developing countries. A tighter lockdown depresses the stock market of developed economies more than the market in developing economies. One reason to explain this differential might be a higher level of compliance with the closure rules in advanced countries. Likewise, stock prices in rich economies respond more strongly to measures of economic support. Again, we find a counterintuitive, positive response of the stock market to restrictions of peoples' mobility. This response is particularly pronounced for developed economies.

Figure 2 (right) documents the impulse responses of NO_2_ emissions across developed and developing countries. The most striking difference cross country groups can be observed for the response of emissions to our proxy for closure policies. In developing countries, we observe a significant drop in emissions after a tightening of the lockdown. In developed countries, in contrast, emissions increase. Hence, the negative response for the full sample, see Figure 1 (right), is driven by the large number of developing countries in our sample. There are two potential explanations for this differential response. First, the sectoral composition of developing economies might be tilted towards manufacturing, that is, emission‐intensive, industries, whereas the service sector dominates in developed countries. Thus, a lockdown that equally depresses both manufacturing production and the services sectors results in a stronger fall in emission in developing countries. This explains why one response is stronger negative than the other. A second explanation offers a reason for why emissions actually increase in richer economies. An important source of NO_2_ emissions is transportation. In advanced economies, a tighter lockdown motivates people to switch from public transportation to individual vehicles, which raises emissions.^11^ This option is not easily available in poorer countries.

### 5.3 Differing income levels

**Figure 3:**
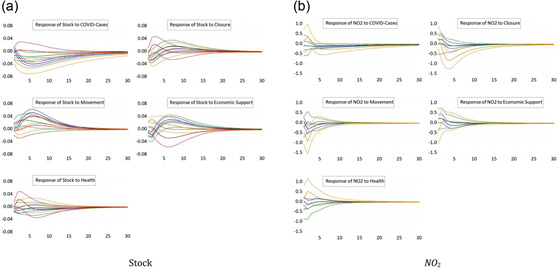
Reaction for different income levels. Impulse responses of stock prices (left) and nitrogen dioxide (NO_2_) emissions (right) to a one‐standard‐deviation shock in coronavirus disease (COVID) cases and the four indicators of government responses. The dashed lines indicate the 95% confidence interval. The blue lines are the responses for high‐income countries, the green lines for upper middle income countries, and the orange lines for lower‐middle income countries. The classification of countries follows the World Bank classification https://datahelpdesk.worldbank.org/knowledgebase/articles/906519. [Color figure can be viewed at wileyonlinelibrary.com]

We now split the sample according to the World Bank's classification of countries' income levels. Although stock prices in high‐income countries fall upon new information about COVID cases in high‐income countries, see Figure 3 (left), they remain unaffected in low‐income countries. Economic support props up the stock market of high‐income and upper‐middle income countries, but remain ineffective with respect to stock prices in lower‐middle income and low income countries. Importantly, the economic support index reflects whether or not national authorities undertook fiscal efforts to stabilize the economy. It does not, however, measure the volume of fiscal policy packages. Hence, the nature and the absolute magnitude of the fiscal interventions strongly differ across countries, which explains why stock markets in poor countries remain insensitive to economic support.

The level of NO_2_ emissions, see Figure 3 (right), also exhibits unequal responses across income levels.^12^ Closing down shops, offices and factories has a particularly strong effect on emissions in lower‐middle income countries, where emissions fall by about 1%. This also implies that an eventual lifting of the lockdown strongly boosts emissions, and hence economic activity, in these countries. In the other income groups, this response is much weaker and often insignificant. A similar picture emerges from the responses to a restriction of the movement of people. This measure is particularly effective in reducing emissions in poorer countries.

### 5.4 Geographical differences

Now we study the regional variation in the impulse responses. For each variable of interest, the figures show the impulse response functions derived for a specific continent against the responses of the rest of the world, that is, the remaining countries.

**Figure 4:**
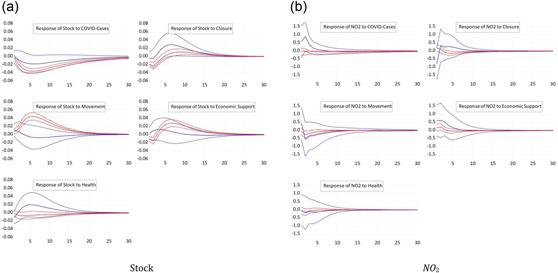
Reaction Africa versus the rest of the world. Impulse responses of stock prices (left) and nitrogen dioxide (NO_2_) emissions (right) to a one‐standard‐deviation shock in coronavirus disease (COVID) cases and the four indicators of government responses. The dashed lines indicate the 95% confidence interval. The blue lines are the responses for African countries, the red lines for the countries in the rest of the world. [Color figure can be viewed at wileyonlinelibrary.com]

**Figure 5:**
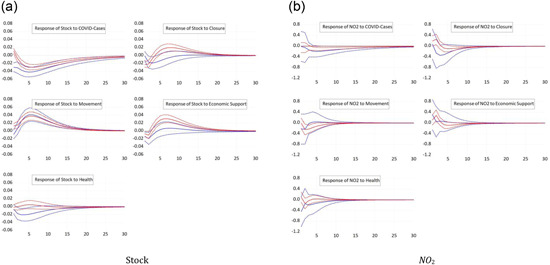
Reaction Asia versus the rest of the world. Notes: Impulse responses of stock prices (left) and nitrogen dioxide (NO_2_) emissions (right) to a one‐standard‐deviation shock in coronavirus disease (COVID) cases and the four indicators of government responses. The dashed lines indicate the 95% confidence interval. The blue lines are the responses for Asian countries, the red lines for the countries in the rest of the world. [Color figure can be viewed at wileyonlinelibrary.com]

The most remarkable difference across countries is the heterogeneity in the responses to economic support packages. In Africa, Asia, and North America, see Figures 4, 5, and 7 (all left), stock prices remain insensitive to economic support measures. In Europe, South America, and Australia, see Figures 7, 8, and 9 (all left), in contrast, we find a significant increase in stock prices after the adoption of economic support measures.

**Figure 6:**
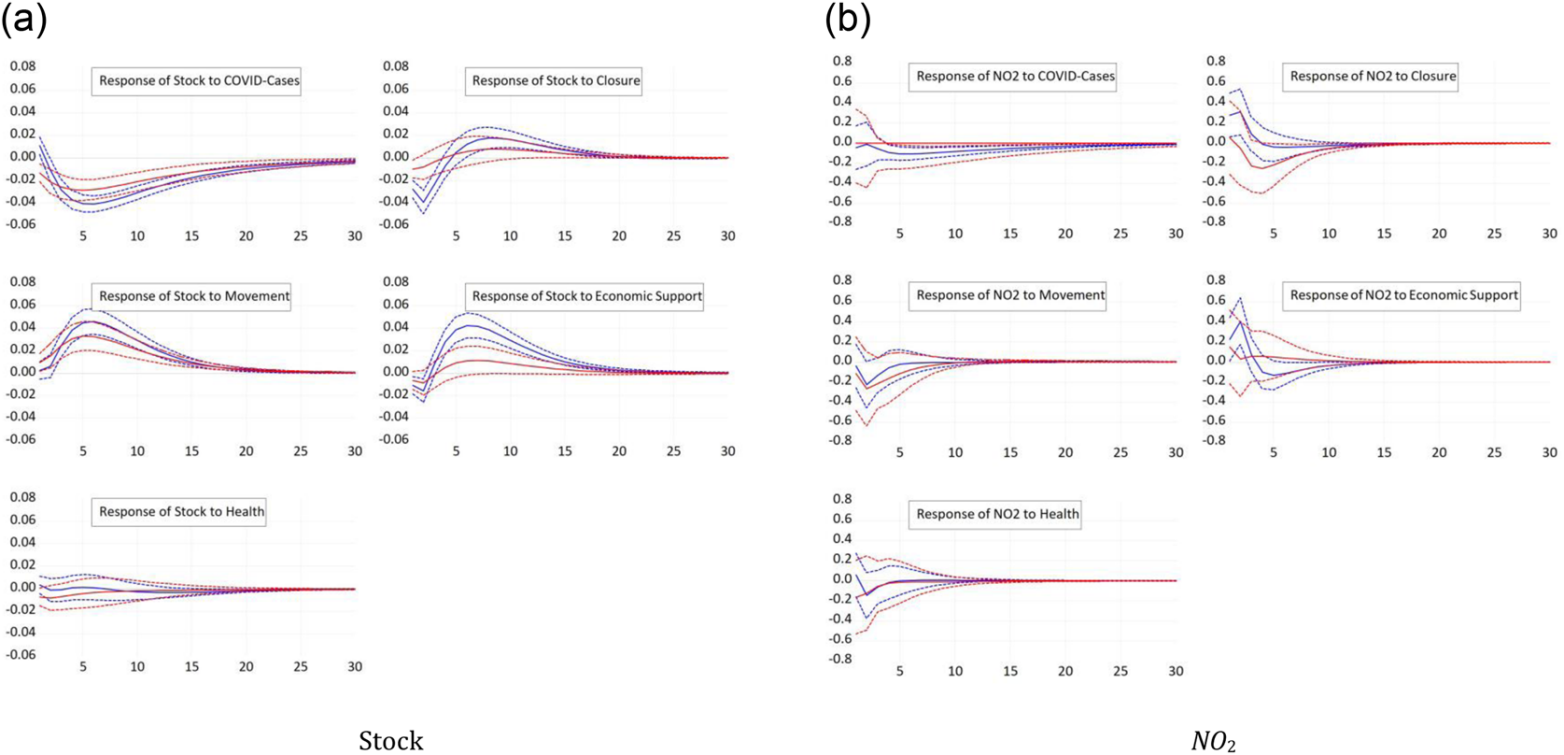
Reaction North and Middle America versus the rest of the world. Impulse responses of stock prices (left) and nitrogen dioxide (NO_2_) emissions (right) to a one‐standard‐deviation shock in coronavirus disease (COVID) cases and the four indicators of government responses. The dashed lines indicate the 95% confidence interval. The blue lines are the responses for North and Middle American countries, the red lines for the countries in the rest of the world. [Color figure can be viewed at wileyonlinelibrary.com]

**Figure 7:**
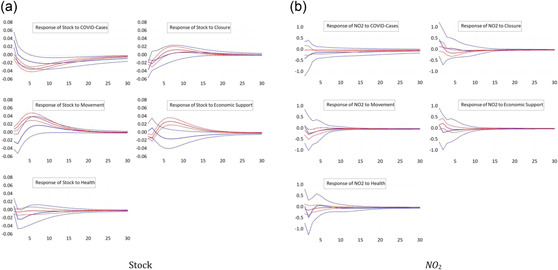
Reaction Europe versus the rest of the world. Impulse responses of stock prices (left) and nitrogen dioxide (NO_2_) emissions (right) to a one‐standard‐deviation shock in coronavirus disease (COVID) cases and the four indicators of government responses. The dashed lines indicate the 95% confidence interval. The blue lines are the responses for European countries, the red lines for the countries in the rest of the world. [Color figure can be viewed at wileyonlinelibrary.com]

**Figure 8:**
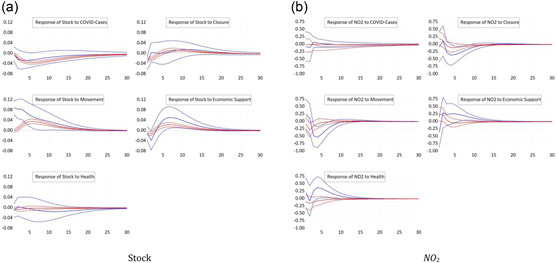
Reaction South America versus the rest of the world. Impulse responses of stock prices (left) and nitrogen dioxide (NO_2_) emissions (right) to a one‐standard‐deviation shock in coronavirus disease (COVID) cases and the four indicators of government responses. The dashed lines indicate the 95% confidence interval. The blue lines are the responses for South American countries, the red lines for the countries in the rest of the world. [Color figure can be viewed at wileyonlinelibrary.com]

**Figure 9:**
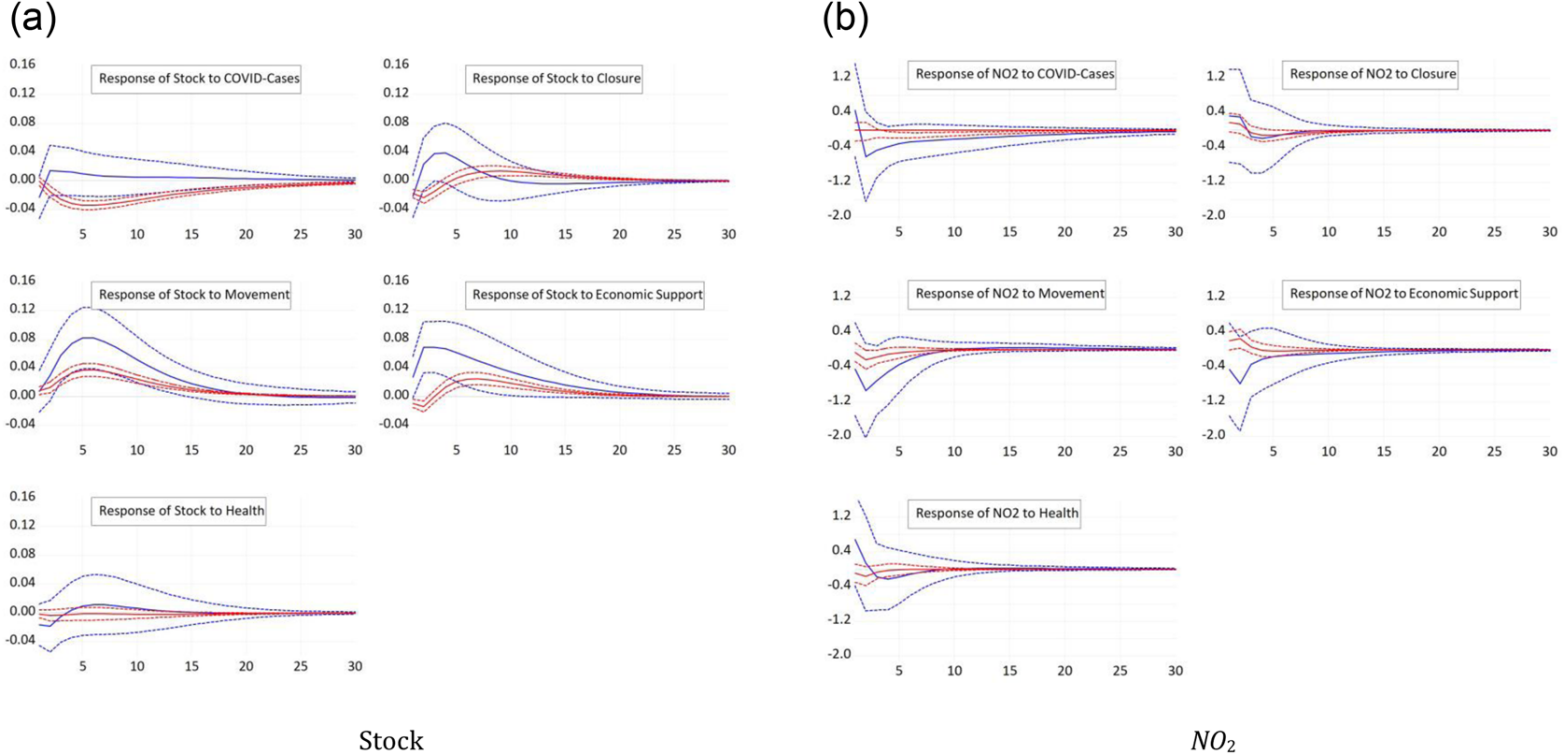
Reaction Australia versus the rest of the world. Impulse responses of stock prices (left) and nitrogen dioxide (NO_2_) emissions (right) to a one‐standard‐deviation shock in coronavirus disease (COVID) cases and the four indicators of government responses. The dashed lines indicate the 95% confidence interval. The blue lines are the responses for Australian countries, the red lines for the countries in the rest of the world. [Color figure can be viewed at wileyonlinelibrary.com]

The positive response of stock markets to economic support measures in Europe is consistent with the positive effect of economic support on European emissions on impact, see Figure 6 (right). In Europe, a higher number of COVID infections reduce emissions—an effect we do not find for most other regions. In South America, see Figure 8 (right), movement restrictions and closures significantly reduce emissions.

### 5.5 The effects across waves

The spread of the pandemic progressed in waves. Policy measures in the first wave of infections might be more or less effective than in the second wave. Although in the first wave policy faced enormous uncertainty about the spread of the virus, the effectiveness of containment policies, and the macroeconomic collateral damage, authorities gathered experience and knowledge over time. Hence, the policy interventions during the second wave could be more precisely targeted, both in terms of timing and scope. As a consequence, the responses of stock markets and emissions to policy interventions could vary over time.

**Figure 10:**
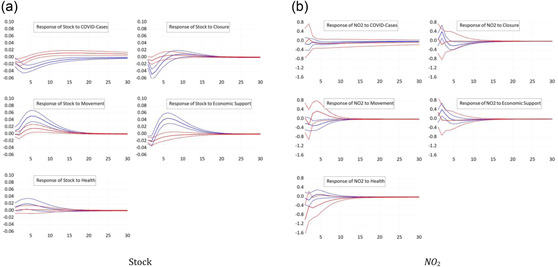
Reaction for different coronavirus disease (COVID) waves. Impulse responses of stock prices (left) and nitrogen dioxide (NO_2_) emissions (right) to a one‐standard‐deviation shock in COVID cases and the four indicators of government responses. The dashed lines indicate the 95% confidence interval. The blue lines are the responses in the first COVID wave, whereas the red lines are those for the second COVID wave. [Color figure can be viewed at wileyonlinelibrary.com]

In Section 3.2, we determined the timing of the COVID waves for each sample country. Importantly, we do not assume that waves occur simultaneously across countries. Such a situation could be captured by a simple sample split. Instead, we identify country‐specific waves before estimating the panel VAR model for the first and the second wave separately. The resulting responses of stock prices are shown in Figure 10 (left). All five impulse responses suggest a common pattern: the responses are much stronger in the first wave compared to the second. Stock prices fall strongly in the first wave as a response to an increase in the number of infections, whereas the drop is much smaller in the second wave.^13^ Likewise, closing down the public life triggers a depreciation of the stock market during the first wave but not the second. The responses to economic support measures across the two waves exhibit a striking difference: during the first wave, supportive policy contributes strongly to an increase in equity market valuation. During the second wave, in contrast, stock prices fall mildly as a response to economic support. The puzzling response of stock prices to movement restrictions is mostly driven by the response during the first wave. Apparently, markets appreciate restrictions of movements as a sign that authorities take the pandemic seriously.

The responses of NO_2_ emissions across the two waves, which are shown in Figure 10 (right), are in line with our expectations. In both waves, the responses of emissions to changes in the number of infections remains insignificant. Closures reduce emissions during the first wave, but not in the second. Put differently, closures significantly constrained economic activity in the first wave, but remained relatively innocuous in the second. The response of emissions to movement restrictions and health measures is consistent with that: restrictions during the first wave significantly reduce emissions, whereas restrictions during the second wave have no significant effect on emissions. With this findings, we are in line the results of Wang et al. (2021) who also find that the effects in the first wave are larger than in the second wave. These results, moreover, suggest that lockdown measures lead to a significant contraction of economic activity. Economic support packages adopted by governments cushioned some of these effects. During the first wave of infections, NO_2_ emissions increase in the first months after new support measures are announced. In the second wave, in contrast, economic support measures remain ineffective in stimulating economic activity as reflected in NO_2_ emissions.

Hence, the impact of policy interventions on both stock prices and emissions are strongly dependent on the state of infections. A policy that is effective during the first wave might no longer be effective in the second wave of infections.

## 6 CONCLUSIONS

In this paper, we estimated the impact of the COVID‐19 pandemic and the policy responses to the pandemic in a large panel of countries. To track the economic impact on a high frequency, we concentrate on the response of stock returns and the growth rate of NO_2_ emissions. These variables are available on a daily frequency, whereas conventional indicators such as industrial production, inflation, and employment are available on a monthly frequency only. Importantly, the large crosssectional dimension allows us to split the sample along the lines of several dimensions and compare the responses across subsamples.

We find that both measures of economic activity are sensitive to the spread of the virus and the policy responses, respectively. A surprise increase in the number of infections triggers a drop in our two measures of economic activity. Both stock returns and NO_2_ emissions fall as a response to closure policies and restrictions of the movements of people. Propping up economic support measures, in contrast, raises stock returns and emissions and, thus, contributes to the economic recovery. These responses strongly differ across subsamples. For example, tightening lockdown measures reduces stock market valuations in developed countries more than in developing countries. In addition, a tighter lockdown significantly reduces emissions in developing countries. Advanced economies, in contrast, exhibit an increase in emissions following a tightening of lockdowns. We also distinguish between the first and the second wave of infections. We find that the responses of stock prices are much stronger in the first wave compared to the second. This finding pertains to the responses to the number of infections and the tightening of lockdowns. The positive impact of economic support measures found in the full sample stems from the first wave only. Consistently, economic support raises emissions in the first wave, but not the second.

Our findings have a number of policy implications: first, the effects of the spread of the virus as well as the containment measures such as closures or movement restrictions on stock prices and NO_2_ emission can be mitigated by expansionary fiscal support. Thus, fiscal stabilization policies are key in dampening the financial and economic impact of the pandemic.

Second, since we have verified that there is considerable heterogeneity across country groups, there is no common recipe to fight the COVID pandemic in all countries. Our results instead suggest that the optimal mix of policy measures to stabilize the economy depends on national characteristics and thus needs to be designed on a country level. This does not mean that countries can and should not learn from the experiences of others. But to do so, at least the degree of development, the income level and geographical properties, among possible other socioeconomic factors, should be taken into account.

Third, the effectiveness of policy measures seems to be time‐varying, that is, the effects are larger in the first wave than in the second. Thus, it can be assumed that policy measures have lower effects the longer the pandemic lasts, or the more waves an economy experiences. This could be interpreted as bad news since stabilization measures need to be bolder in late waves to have the same quantitative effect than in earlier waves. However, it could also be seen as good news since we have also shown that the response of activity to the COVID pandemic is also reduced for later waves. Thus, the need for stabilizing measures should be lower, as the economies seem to learn how to live with the COVID pandemic.

Nevertheless some limitations of our study remain. First, governments did not only impose fiscal policy measures to fight the economic effects of the COVID crisis. In addition, monetary policy intervened in most countries. Therefore, it would be important to add those policy responses in a separate indicator. Even more, fiscal and monetary policy used very different tools to stabilize the economy. It could be investigated to what extent the effectiveness of those instruments differs. Second, we chose one financial and one real variable to show the effects. However, it is not guaranteed that all financial and real variables react in the same fashion as stock prices and NO_2_ emissions. Therefore, it should be checked to what extent our results can be generalized to other financial and real variables. We leave both research questions for further research.

## Data Availability

The data that support the findings of this study are openly available at https://sites.google.com/site/profjensklose/data

## Footnotes

1 Ludvigson et al. (2020) follow a similar approach to Baker, Bloom, and Terry (2020) by focusing on the impact of a disaster in the United States on uncertainty and economic performance in a VAR framework. Concerning uncertainty, they use three different measures as follows: first, macroeconomic uncertainty (Jurado et al., 2015); second, financial uncertainty (Ludvigson et al., 2019); and, third, economic uncertainty (Baker et al., 2016).

2 Of course, there are other studies focusing on the response of other financial market variables in the wake of the COVID pandemic. See, for example, Klose and Tillmann (2021), Zaremba et al. (2022), or Ali et al. (2022), with respect to bonds, Aharon et al. (2022) with respect to yield curves, Umar, Aziz, et al. (2021) or Esparcia et al. (2022) with respect to precious metals, or Umar, Jareno, et al. (2021) as well as Klose (2022) with respect to currencies.

3 An alternative would be the use of daily country data for CO_2_ emission. To the best of our knowledge, this data is not available on the frequency and for the set of countries we need it. Moreover, indirect measures like the one of Mzoughi et al. (2020) cannot be applied in our context, since individual country data are not available.

4 Since we explicitly model only the period since the outbreak of the COVID pandemic, the critique to the use of VAR models in this context as recently expressed by Lenza and Primiceri (2020) or Ng (2021) does not apply to our analysis.

5 See https://datahelpdesk.worldbank.org/knowledgebase/articles/906519.

6 Goldstein et al. (2021) show that containment measures loose their effectiveness with respect to the spread of the pandemic after about 4 months. Hence, the effect of lockdowns is unstable over time. The authors refer to “lockdown fatigue” to characterize this finding.

7 Finck and Tillmann (2022) use a smooth‐transition model to differentiate between different stages of the pandemic.

8 With this definition, we are a bit less restrictive than the British Office for National Statistics (2021) who use a 15‐day period of continuous positive growth rates. Our approach allows us to identify more short‐lived waves, for example, seen in Australia.

9 Since the United States is the only country with three waves identified in our sample period, we cannot estimate the effects of a third wave properly. Therefore, we do not use the information of the third wave in the United States but only compare the effects of the first with those of the second wave that most countries experienced.

10 Results for the third category, the transition countries, have been excluded due to large confidence bands because of the low number of observations. Those results are, however, available from the authors upon request.

11 However, Aruga et al. (2022) find that CO_2_ emission decreases in the US and France in response to lower mobility, which can be associated with closures. However, there may be several reasons for the differences in their and our results. First, we focus on NO_2_ instead of CO_2_. Second, we rely on a broader set of countries and a considerably longer time period. Third, the Google indicator used by Aruga et al. (2022) does not differentiate between private and public transport. Thus, tighter lockdown measures can in fact result in higher or lower private mobility (e.g., via using own cars), thus raising or decreasing emission.

12 It is noteworthy that NO_2_ data for low income countries are not available, so no estimation results can be presented for this group of countries.

13 The finding of a negative response of stock prices to the spread of the virus is consistent with Yousfi et al. (2021) who find the same pattern for the US stock market but do not quantify in which wave the effects were stronger.
